# Application of reinforcement learning for segmentation of transrectal ultrasound images

**DOI:** 10.1186/1471-2342-8-8

**Published:** 2008-04-22

**Authors:** Farhang Sahba, Hamid R Tizhoosh, Magdy MA Salama

**Affiliations:** 1 Medical Instrument Analysis and Machine Intelligence Group, University of Waterloo, Waterloo, Canada; 2 Department of Systems Design Engineering, University of Waterloo, Waterloo, Canada; 3 Department of Electrical and Computer Engineering, University of Waterloo, Waterloo, Canada

## Abstract

**Background:**

Among different medical image modalities, ultrasound imaging has a very widespread clinical use. But, due to some factors, such as poor image contrast, noise and missing or diffuse boundaries, the ultrasound images are inherently difficult to segment. An important application is estimation of the location and volume of the prostate in transrectal ultrasound (TRUS) images. For this purpose, manual segmentation is a tedious and time consuming procedure.

**Methods:**

We introduce a new method for the segmentation of the prostate in transrectal ultrasound images, using a reinforcement learning scheme. This algorithm is used to find the appropriate local values for sub-images and to extract the prostate. It contains an offline stage, where the reinforcement learning agent uses some images and manually segmented versions of these images to learn from. The reinforcement agent is provided with reward/punishment, determined objectively to explore/exploit the solution space. After this stage, the agent has acquired knowledge stored in the Q-matrix. The agent can then use this knowledge for new input images to extract a coarse version of the prostate.

**Results:**

We have carried out experiments to segment TRUS images. The results demonstrate the potential of this approach in the field of medical image segmentation.

**Conclusion:**

By using the proposed method, we can find the appropriate local values and segment the prostate. This approach can be used for segmentation tasks containing one object of interest. To improve this prototype, more investigations are needed.

## Background

Ultrasound imaging is one of the most widely used technologies for diagnosis and treatment. These images are the result of reflection, refraction and deflection of ultrasound beams from different types of tissues with different acoustic impedance [1]. Some factors, such as poor contrast, speckle and weak edges, however, make these images a challenging case for segmentation. Further complications arise when the quality of the image is influenced by the type and settings of equipment. The prostate segmentation of TRUS images is a well-known case study [1,2]. The detection of the prostate boundary in such images is crucial for automatic cancer diagnosis and classification. However, due to a very low signal-to-noise ratio, it is difficult to extract all of the correct boundaries. Hence, any improvements in the segmentation process is desirable. Many methods have been introduced in literature to facilitate more accurate automatic or semi-automatic segmentation of the prostate boundaries in ultrasound images [3-11]. The performance is usually improved by taking expertise or priori knowledge into account. Generally, all segmentation methods require at least some user interaction to adjust critical parameters. The type of user interaction varies, depending on the amount of time and effort required from the user.

By studying the existing methods, we can observe that they may require many training samples if they rely on learning techniques, or that some user interactions are necessary to determine the initial values. Also, many methods cannot improve their performances through time. Considering these factors, a new algorithm based on reinforcement learning (RL) is proposed. Several approaches have already been introduced, which show the application of RL for image-based problems [12-18]. In our algorithm, we use the reinforced adjustment to control the local processing parameters for the segmentation of the prostate in TRUS images. The goal is to propose an approach that has the following characteristics:

• it requires a limited amount of training data

• it improves performance with continuous feedbacks

It is important to note that our proposed approach is not designed to compete with the existing segmentation approaches. The aim of this work is the proof of concept, by presenting a prototype of such an approach.

Due to the nature of RL, in terms of the state, action and reward definitions and their interactions with each other, this approach can acquire knowledge and adapt this knowledge according to new input images. It learns in two modes: offline and online. It recognizes the parameters for all processing stages through exploratory learning in the offline mode. Then, this information is exploited during the online mode, where the approach simultaneously modifies its knowledge. The structure used in this approach can also incorporate subjective evaluation as a feedback. The final goal is to identify the object of interest in an image.

### Reinforcement learning

Reinforcement learning (RL) is derived from the idea that an agent learns the correct behavior through interactions in a dynamic environment [19]. The history of RL can be traced to the solution of optimal control problem, by using value functions and dynamic programming [20]. The agent automatically determines the ideal behavior within a specific context that maximizes performance with respect to predefined measures. The RL agent, the decision maker of the process, observes the state of the environment and takes an action that influences the environment. This action is based on the former experience, associated with the current observation and accumulated reinforcement, a reward or punishment. Reward or punishment is determined from the environment, depending on the action taken. The RL agents discover the optimal policy for decision-making through *exploration *and *exploitation*. In the exploration stage, the agent attempts to discover which actions yield the maximum reward by taking different actions repeatedly, whereas in the exploitation stage, it selects the actions that yield more rewards. The agent also receives information concerning the state of the environment. At the beginning of the learning process, the RL agent does not have any knowledge of the result of choosing different actions. The agent takes various actions and observes the results. After a while, the agent explores many actions that bring the highest reward and gradually exploits them. In fact, it acquires knowledge from the actions and eventually learns to perform those that are the most rewarding. During this process, the agent tries to meet a certain goal with respect to the optimal state. The reward and punishment are defined objectively when they are based on desired properties of the results, or gained subjectively, when the agent receives them directly from the interactive user.

The action policy, *π*, in RL is the strategy used by the agent to select an action to change the current state. The agent must find a trade-off between immediate and long-term returns. It must explore the unseen states, as well as the states which maximize the return by choosing what the agent already knows. Therefore, a balance between the exploration of unseen states and the exploitation of familiar (rewarding) states is crucial. Watkins has developed Q-learning, a well-established on-line learning algorithm, as a practical RL method [21,22]. In this algorithm, the agent maintains a numerical value for each state-action, representing a prediction of the worthiness of taking an action in a state. Table 1 represents an iterative policy evaluation for updating the state-action values where *r *is the reward value received for taking action *a *in state *s*, *s' *is the next state, *α *is the learning rate, and *γ *is the discount factor [19,20,23]. There are some policies for taking action *a *given state *s*. One of them is the Boltzman policy which estimates the probability of taking each action in each state. There are other policies for Q-learning such as *ε*-greedy and greedy. In the greedy policy, all actions may not explored, whereas the *ε*-greedy selects the action with the highest Q-value in the given state with a probability of 1 – *ε*, and other ones with a probability of *ε *[20,23]. In this work an *ε*-greedy policy is used to make a balance between exploration and exploitation in the offline and online modes.

**Table 1:** Q-Learning algorithm

Initialize *Q*(*s*, *a*) arbitrary
Repeat (for each episode):
Initialize state *s*
Repeat (for each step of episode):
Choose action *a *from state *s *using policy derived from *Q *(e.g., *ε*-greedy)
Take action *a*, observe reward *r*, next state *s'*
$Q(s,a)\leftarrow Q(s,a)+\alpha [r+\gamma \underset{a^{\prime }}{\max}Q(s^{\prime },a^{\prime })-Q(s,a)]$
*s *← *s'*;
until *s *is terminal

The reward *r*(*s*, *a*) is defined according to each state-action pair (*s*, *a*). The goal is to find a policy to maximize the discounted sum of rewards received over time. The principal concern in RL are the cases where the optimal solutions cannot be found, but can be approximated. The online nature of RL distinguishes it from other techniques that approximately solve Markov decision processes (MDP)[19,23]. Ideally, the RL agent does not require a set of training samples. Instead, it can continuously learn and adapt while performing the required task. Although this is not a supervised learning, a reward function is employed, so that a weak supervision is assumed. This behavior is desirable in many cases, where a sufficient number of precise learning samples is difficult or impossible to obtain.

In this paper, we attempt to introduce a segmentation approach based on an RL agent. This agent is goal-oriented, which attains the highest reward by finding the optimal parameters for the different processing stages.

## Methods

**Figure 1:**
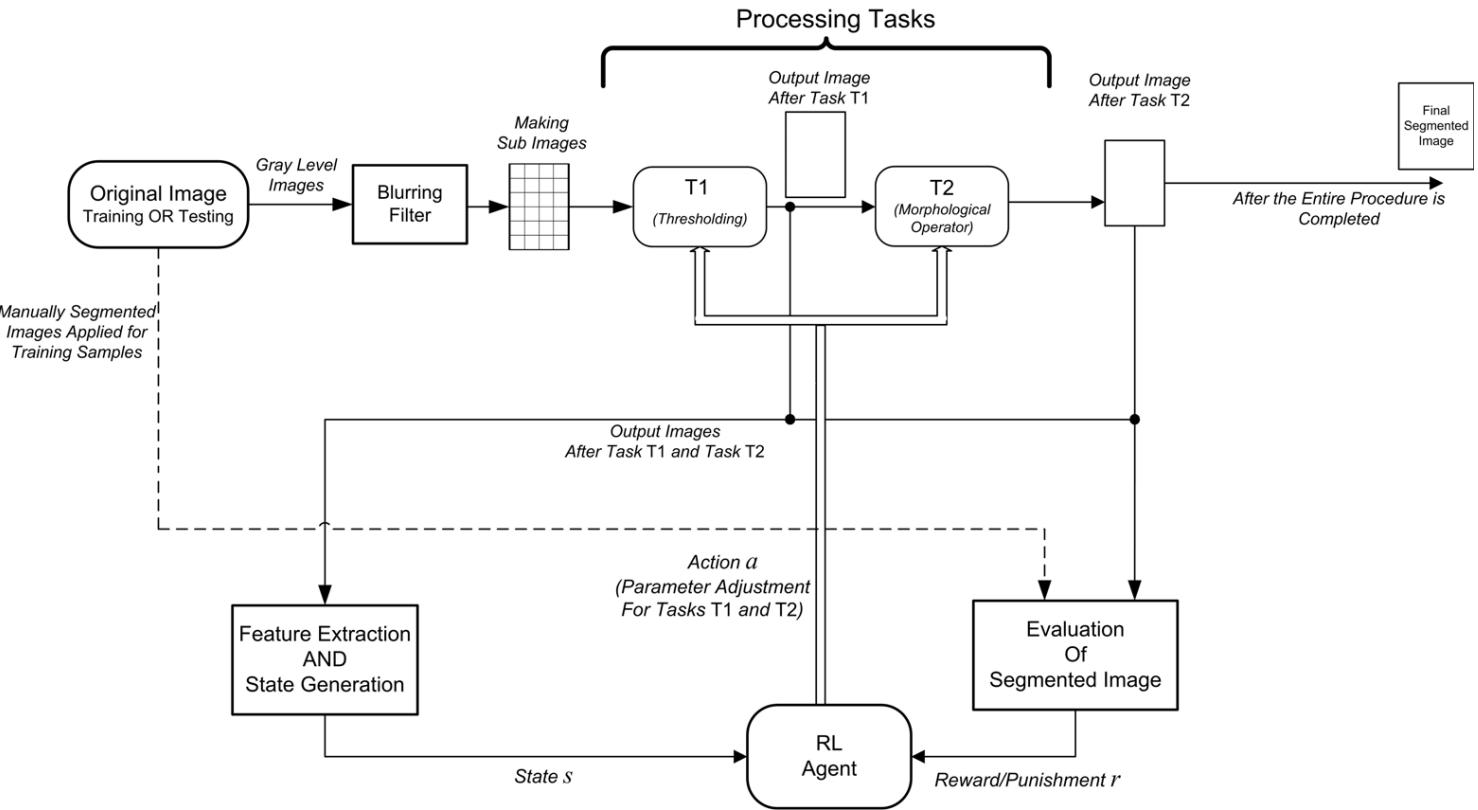
The proposed approach for reinforced ultrasound image segmentation.

A framework for adjusting the parameters of a two-stage segmentation approach, by using an RL agent, is introduced in this section. The framework is depicted in Figure 1. As shown, an intelligent agent is employed to find the appropriate parameters for processing tasks and segment the input images. Each processing task has parameters that must be adjusted. The goal is to choose a proper set of parameters for various tasks, such that an object of interest is extracted.

An RL layout is required where the states are the features that describe the image in various stages. Also, the actions have the capability to change the corresponding parameters. The reward can be evaluated based on the quality of the output image.

### Design aspects

As shown in Figure 1, the approach contains two image processing tasks {*T*_1_, *T*_2_} with specific parameters. It adaptively computes the set of parameters that extract the object of interest. Limiting ourselves to the model described in the previous section, we must first define the states *s*, actions *a *and reward *r*. To generate the state, features that describe the objects in the image after each processing task are extracted. The actions are proper adjustments of the parameters of the processing tasks. After the agent changes these parameters, it receives a reward which is an external signal provided by the environment and must correctly reflect the goal. When the agent modifies the parameters of the processing tasks, an evaluation metric is used to assess the result and produce a reward. The details about how three components, namely state, action, and reward are defined in our proposed approach are described later (see the next subsections).

The RL approach for prostate segmentation has two modes: offline and online. Offline training is conducted by using the manually segmented samples represented as " ground-truth images ". In this mode, the agent is adopted in a simulation environment and interacts with training samples to acquire information about the required parameters. Once the agent's behavior is acceptable in terms of low dissimilarities between the ground-truth images and the results of the approach, the agent switches to the online mode. In this mode, the RL agent is used directly in a real-time situation with new images. With the agent learning continuously in this mode, it can adapt to the changes in the background and object. In this mode, the RL agent applies its knowledge acquired during the offline mode and applies it on new images. Considering the RL nature, the learning stage can be performed by using a limited number of training samples and gaining more knowledge during the online mode.

The inputs are TRUS images. A median filter (7 × 7) is applied, first to smooth the input image *I*. This image is then divided into *R*_*I *_× *C*_*I *_sub-images (for *R*_*I *_rows and *C*_*I *_columns). The RL agent works on each of them separately. It continues until a criterion is met or a maximum number of iterations is reached. Local processing on sub-images is carried out to find the best segmentation parameters for each of them. To make a binary image, the sub-images are thresholded using local threshold values in task *T*_1_. Due to disturbing factors such as poor contrast, noise, or non-uniform illumination, artifacts exist after thresholding. Therefore, morphological opening with a disk-shaped structuring element is applied as task *T*_2 _to postprocess each thresholded sub-image and remove the artifacts. The RL agent determines the local threshold values and the size of the structuring elements for each sub-image.

During the offline training, where the desired output image is available, the agent works on each sub-image and explores the solution space. Dissimilarity, $D_{i_{sim}}$, between the result of the approach for sub-image *i*_*sim *_and its desired (ground-truth) output $i_{sim_{d}}$ is defined as the normalized number of misclassified pixels:

(1) $$D_{i_{sim}}=\frac{XOR(i_{sim},i_{sim_{d}})}{r_{sim}\times c_{sim}}$$

where *XOR *indicates the number of mismatched pixels between these two sub-images, and *r*_*sim *_and *c*_*sim *_are the number of rows and columns for the sub-images. The agent continues until the dissimilarity is less than a pre-defined value *ϑ*. In this mode, the agent tries different actions in different states with an exploratory procedure. After the RL agent changes the parameters for each sub-image, the agent receives a corresponding reward for that state-action pair and updates the corresponding value in the Q-matrix. By the end of offline mode, the agent has explored many actions and then tries to exploit the most rewarding ones during the following online mode where no ground-truth images are available.

**Figure 2:**
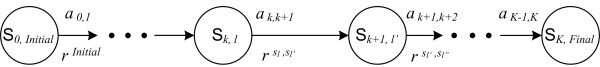
State transition model for a sub-image.

In the online mode, the agent works on each sub-image and completes the process of segmentation by using the knowledge it has previously gained. The rewards are not calculated immediately, and the agent waits until the whole image is scanned. Then, the reward is provided for each sub-image based on the quality of segmented object before and after the action taken. The state transition diagram for a sample sub-image is shown in Figure 2. In this diagram, *s*_*k*,*l *_indicates the state number *l *in iteration *k*. The action *a*_*k*,*k*+1 _is one of the possible actions that changes the current state *s*_*k*,*l *_to the next state *s*_*k*+1,*l*' _and receives the reward $r^{s_{l},s_{l^{\prime }}}$. In this diagram, *s*_*l*_, *s*_*l*' _and *s*_*l*" _represent arbitrary states. For each sub-image, the agent starts from initial state *s*_0,*initial *_and after *K *iterations, it arrives at the final (desired) state *s*_*K*,*Final*_. To construct the RL agent, three components: states, actions and rewards, should be defined in the following subsections.

### State definition

The idea of the state containing features, which represent the status of the binary image after each step of the processing task is considered as

(2) *s *= [*χ*_1 _*χ*_2 _*χ*_3 _*χ*_4_],

where *χ*_*i *_is the selected feature. The features reflect the local segment status after different processing tasks. Due to applying the approach to each sub-image, employing features which result in an extremely large state space or significantly increase the computational complexity is not desirable. Instead, the states must simply represent the features of the objects in the binary image for each sub-image. This is a fundamental characteristics of our proposed approach.

For prostate segmentation in TRUS images, considering the above conditions (representing the local status of the revealed objects after the processing tasks), the following features in each sub-image are extracted to define the states:

#### Area

In each sub-image, the area of the largest object, *A*_*object*_, is chosen to define the state. The normalized area with respect to the whole area of the sub-image is calculated and used as the first feature. It is represented as

(3) $$\chi_{1}=\frac{A_{subimage}-A_{object}}{A_{subimage}}$$

#### Compactness

The compactness of the largest object after thresholding defined as

(4) $$\chi_{2}=\frac{4\pi A}{P^{2}}$$

is adopted as the second feature to represent the state. where *A *and *P *are the area and perimeter of the object in the sub-image, respectively [24].

#### The location of each sub-image

The image is divided into several sub-images and the cartesian coordinates of each sub-image is used as a state parameter to indicate on which region of the image the approach is working.

(5) *χ*_3 _= (*x*_*s*_, *y*_*s*_)

#### Number of the objects

The last parameter used in state definition is the number of revealed objects, *N*_*O*_, after the morphological opening. This feature helps to indicate if the size of structuring element is lower or higher than appropriate value.

(6) *χ*_4 _= *N*_*O*_

### Action definition

To extract the object of interest, the actions are defined as changing of the parameters of the processing tasks for each sub-image. The assigned local threshold value *τ*_*i *_is increased or decreased by a specific amount *δ *from equally spaced values (*τ*_1_, *τ*_1_, ..., *τ*_*n*_) between the local maximum gray level *g*_*lmax *_and local minimum gray level *g*_*lmin *_in each iteration. For morphological operator the agent chooses the size of structuring element *v*_*j *_among predefined values (*v*_1_, *v*_2_, ..., *v*_*m*_) to control its effect. As a result, there exist a set of actions, A, as follow:

(7) $$A=a_{1}\cup a_{2}$$

where *a*_1 _and *a*_2 _represent the actions that change the parameters of the processing tasks *T*_1 _and *T*_2_, respectively. Using this definition, a binary image using the local threshold values *τ*_*i *_and local structuring element *v*_*j *_is obtained. Thus, the set of all actions A can be presented as follow:

(8) $$A=\{\tau_{i}\pm \delta \}\cup \{v_{j}\},$$

where *τ*_*i *_∈ [*g*_*lmin*_, *g*_*lmax*_], *v*_*j *_∈ [*v*_1_, *v*_*m*_], *i *∈ {1, .., *n*}, *j *∈ {1, .., *m*}.

### Reward definition

The rewards and punishments are defined according to the quality criterion that represents how well the object is segmented in each sub-image. For each sub-image, the agent must receive the reward for improving the quality of segmented object. The reward/punishment must correctly reflect the goal of the system. A straightforward method is comparing the result of the approach with the ground-truth image (manually segmented image) after each action. To measure this value for each sub-image, we note how much the quality has changed after the action. In each sub-image, for improving the quality of segmented object, the agent receives a reward, otherwise it is punished. A general form for the reward function in the offline mode is represented by

(9) $$reward=\{\begin{array}{rr} R_{1} & D_{i_{sim-AFTER}}<D_{i_{sim-BEFORE}},\\ R_{2} & otherwise. \end{array}$$

where $D_{i_{sim-AFTER}}$ and $D_{i_{sim-BEFORE}}$ indicate the dissimilarities with respect to the ground-truth image before and after taking the action, respectively. *R*_1 _and *R*_2 _are constant values.

### Offline learning mode

In the offline mode the ground-truth images segmented by the expert are available. The RL agent works on each sub-image individually. Based on the segmentation quality after each action, the agent receives an objective reward or punishment *r*_*o *_for the sub-image and updates its knowledge. For the quality measure of each sub-image, the dissimilarity $D_{i_{sim}}$ with respect to the ground-truth image is calculated. During this procedure, the agent must explore the solution space by using *ε*-greedy policy with a large value of *ε *[19]. The agent attempts different actions in different states (explained in the previous subsections) by using an exploratory policy. By the end of offline iterations, the Q-matrix is filled with suitable values and the agent can estimate the appropriate action for each given state for new images.

### Online mode

For new samples, where no ground-truth images are available, the agent takes action according to the knowledge it has previously gained. The RL agent finds the appropriate local parameters such that the object of interest can be correctly segmented. In this mode, in each iteration the RL agent finds the parameters for each sub-image and then moves to the next sub-image. This continues until the whole image is scanned. After that, the object contour is considered in a global manner to identify the image regions that correspond to the object of interest. The global model matching method is used to detect the difference between the extracted object and the shape of the object of interest. The RL agent then uses these feedbacks as rewards/punishments for the action taken on each sub-image. If a sub-image is segmented correctly, in terms of the global feedback, the parameters remain unchanged and its process is terminated. The online learning procedure is performed by the following evaluations.

#### Objective evaluation

Objective evaluation is applied as a primary assessment. There are several methods for implementing this evaluation. One way is to use the signature of the extracted object and compare it to the standard signature of the object we are looking for. A signature is a functional representation of a contour [25,26]. Using the geometric center of the prostate in original image, the distance versus angle is measured. To indicate the central point there are two options:

- Using the method introduced by authors in [27] which is an automatic technique.

- Using a manually entered point by the user.

Although both above options can be used, due to the importance of the accuracy and reliability of the central point for the approach, and also to avoid complexity, we preferred to use the manual method in this version of the work (our experiment shows that the automatic method may fail in a few cases). We calculate the distance between the object center and the points located on the boundary as a 2*π *periodic function. In this case, the angle *θ *is assigned to a distance *d *which is the nearest corresponding contour point. It is represented as a function *d *= *f*(*θ*) to generate the central distance signature. We normalize *d *to make this transformation scale-invariant. The standard signature is extracted by using the ground-truth images. Using this information irregular parts are found and used to update the RL agent for new images [17]. The abrupt changes in the signature path (corresponding to the irregular parts) must be detected. To find the points, an estimator based on Kalman filter is used to evaluate the object signature and detect the existence of the attached and/or missing parts [28]. To implement such a technique, we simulate the problem of signature tracing as a dynamic tracking system. In this system the data located on the signature of the segmented object are used as the input (measurement data) for the tracking filter. Using such an estimator the Kalman filter can track the trajectory of the signature for a whole period. Each data on the signature brings information simulated as a dynamic movement. For this movement we must use some variables to describe it. Two simple cases are considered as the position and velocity of the movement. Using this method we can estimate the abrupt changes related to the irregular parts on the border of the segmented object. Finally, the significant deviations are estimated and the objective reward (/punishment) *r*_*o *_for the corresponding sub-image is provided.

#### Subjective evaluation

Another alternative for assessment is subjective evaluation via online feedback. The user considers the results of the segmentation approach for each image. If he/she is not satisfied, he/she can change the results manually. These changes are evaluated as subjective punishment for the agent, where the manually corrected result is used as a new ground-truth image to improve the agent's knowledge. From the global perspective, this evaluation may tend to be biased in favor of the user. Then, the agent processes the next image with its updated information. By adopting this method, the agent is further trained online by a subjective evaluation. Then the Q-matrix is updated and the segmentation can follow the changes in the new input image. During all the previous stages, the object of interest (in our case the prostate) is extracted by using the position of the central point. Some standard processes for boundary refinement may be applied on the extracted object to have a well-shaped final result. The RL agent uses the corrected result as a new ground-truth image to update its knowledge.

## Results and discussion

The proposed approach is implemented using TRUS image slices. The data set contains 60 images with specific prostate shape. To train the agent in the offline mode, eight manually segmented images from the data set were used and the agent was trained. The size of the TRUS images are 468 × 356 pixels. Considering the size of the prostate in TRUS images, we empirically choose *R*_*I *_= 20 and *C*_*I *_= 14. The number of discrete levels for state-action pairs for each individual sub-image was set to 140. The threshold action is defined by taking 15 predefined values (equally spaced with *δ *= 1/15) between the local maximum and minimum gray levels in each sub-image. For the postprocessing action, we chose the size of disk-shaped structuring element among values 0, 2 and 5.

**Figure 3:**
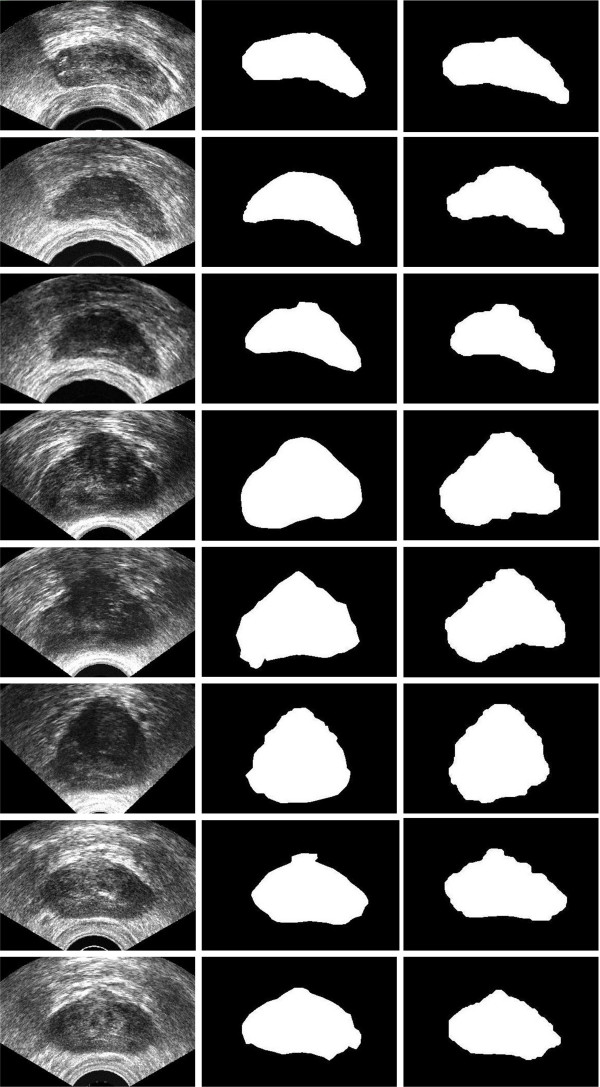
From left to right: original images, ground-truth image (manually segmented), and the results of the proposed approach for eight sample images.

The RL agent was trained using the eight ground-truth images with a standard Q-learning. The criterion to terminate the process for each sub-image is to reach a pixel dissimilarity less than *ϑ *= 5% comparing to the ground-truth image. The values of learning *α *for reinforcement agent was set to 0.8. The average time for training using samples was 94 *s*. We calculate the reward/punishment based on *R*_1 _= 10, *R*_2 _= 0 (see equation 9). After performing the training the Q-matrix is filled with appropriate values. In fact, the agent gained enough knowledge to recognized the optimal values for each sub-image. The approach was applied on the remaining images. The average time for test samples was 8.4 *s*. Figure 3 shows the results for the proposed approach for two training and six testing images.

The choice of criteria to measure the accuracy is dependent on the application and can be derived from the region or boundary information. For all images the accuracy in the final segmented object is defined as *Area Overlap*, *AO*, which is a commonly used Area-based metric for this purpose. Using the terms *TP *(True Positive), *FP *(False Positive), and *FN *(False Negative) for the classified pixels in the result of the approach, it is calculated as the proportional area correctly identified by the approach:

(10) $$AO=\frac{TP}{TP+FN}\times \%100$$

Table 2 shows these results for training images and table 3 shows them for all images including training and testing with their mean and standard deviation.

**Table 2:** The mean *μ *and standard deviation *σ *of Area Overlap (AO%) for training images.

**Image**	**AO(%)**
*1*	90.21
*2*	92.72
*3*	90.63
*4*	95.11
*5*	89.42
*6*	91.86
*7*	89.67
*8*	88.09
*μ*	**90.96**
*σ*	**2.20**

**Table 3:** The mean *μ *and standard deviation *σ *of Area Overlap (AO%) for all training and testing images.

	**AO (%)**
*μ*	88.1
*σ*	3.2

Considering the results in terms of the visual appearance and the analysis presented in [2], the segmentation approach can be employed as suitable coarse level segmentation. This coarse version can then serve a fine-tuning segmentation algorithm to achieve more accurate results. For instance, these results can be used as first level segmentation in the methods introduced by authors in [27].

By using the RL agent in the offline and online modes, the proposed method can find the appropriate local values for two image processing tasks and segment the prostate. Due to the nature of a reinforcement learning agent, in terms of state, action and reward and their interactions with each other, this model is able to adapt to the slight changes in the prostate and background characteristics.

## Conclusion

This paper intended to present the concept of reinforcement learning in the field of TRUS image segmentation, by showing some preliminary results. First, the image was divided into several sub-images. Then in an offline stage, the agent used the ground-truth images as training samples and took specific actions (i.e. changing the threshold value and the size of structuring element) to change its environment (the quality of the segmented parts) in each sub-image. The agent can acquire the necessary knowledge using these samples and fill the Q-matrix after the offline stage. Using this knowledge, the agent took actions with the maximum rewards for each sub-image. Then it switched to the online mode, where the new images are presented. Due to using of the knowledge obtained from the previous input images, this approach is specifically useful where input images have widely similar object and background characteristics. The agent can choose the appropriate parameters values for these images based on its accumulated knowledge. The proposed approach can be used for segmentation tasks containing one object of interest, such as prostate segmentation in TRUS images.

To improve this prototype and achieve more accurate results, the following investigations can be concluded:

• There is a trade-off between the learning time and accuracy in general, a comprehensive study in this regard must be done.

• Extension of the algorithm including the reduction of the training time, and integration of more effective features, including the gray level information for each sub-image must be investigated.

• More actions as well as other combination of processing tasks are subjected to further research.

• As the first version of this work, we have chosen the parameters empirically to introduce the concept and show some preliminary results. Automatic selection of the most parameters must be investigated for the next versions.

## Competing interests

The authors declare that they have no competing interests.

## Authors' contributions

FS developed the main parts of the approach. HRT provided guidance and was involved in the theoretical aspects of algorithm development. MMAS provided guidance. All authors read and approved the final manuscript.

## Pre-publication history

The pre-publication history for this paper can be accessed here:


